# The First Step of *Neurospora crassa* Molybdenum Cofactor Biosynthesis: Regulatory Aspects under N-Derepressing and Nitrate-Inducing Conditions

**DOI:** 10.3390/microorganisms8040534

**Published:** 2020-04-07

**Authors:** Simon Wajmann, Thomas W. Hercher, Sabine Buchmeier, Robert Hänsch, Ralf R. Mendel, Tobias Kruse

**Affiliations:** 1 TU Braunschweig, Institute of Plant Biology, 38106 Braunschweig, Germany; s.wajmann@tu-bs.de (S.W.); t.hercher@tu-bs.de (T.W.H.); r.haensch@tu-bs.de (R.H.); r.mendel@tu-bs.de (R.R.M.); 2 TU Braunschweig, Institute of Physical and Theoretical Chemistry, Antibody Facility, 38106 Braunschweig, Germany; s.buchmeier@tu-bs.de; 3 Center of Molecular Ecophysiology (CMEP), College of Resources and Environment, Southwest University No. 2, Tiansheng Road, Beibei District, Chongqing 400715, China

**Keywords:** molybdenum cofactor, molybdenum cofactor biosynthesis, regulation, *Neurospora crassa*

## Abstract

Molybdenum cofactor (Moco) is the active site prosthetic group found in all Moco dependent enzymes, except for nitrogenase. Mo-enzymes are crucial for viability throughout all kingdoms of life as they catalyze a diverse set of two electron transfer reactions. The highly conserved Moco biosynthesis pathway consists of four different steps in which guanosine triphosphate is converted into cyclic pyranopterin monophosphate, molybdopterin (MPT), and subsequently adenylated MPT and Moco. Although the enzymes and mechanisms involved in these steps are well characterized, the regulation of eukaryotic Moco biosynthesis is not. Within this work, we described the regulation of Moco biosynthesis in the filamentous fungus *Neurospora crassa,* which revealed the first step of the multi-step pathway to be under transcriptional control. We found, that upon the induction of high cellular Moco demand a single transcript variant of the *nit-7* gene is increasingly formed pointing towards, that essentially the encoded enzyme NIT7-A is the key player for Moco biosynthesis activity in *Neurospora*.

## 1. Introduction

**Figure 1:**
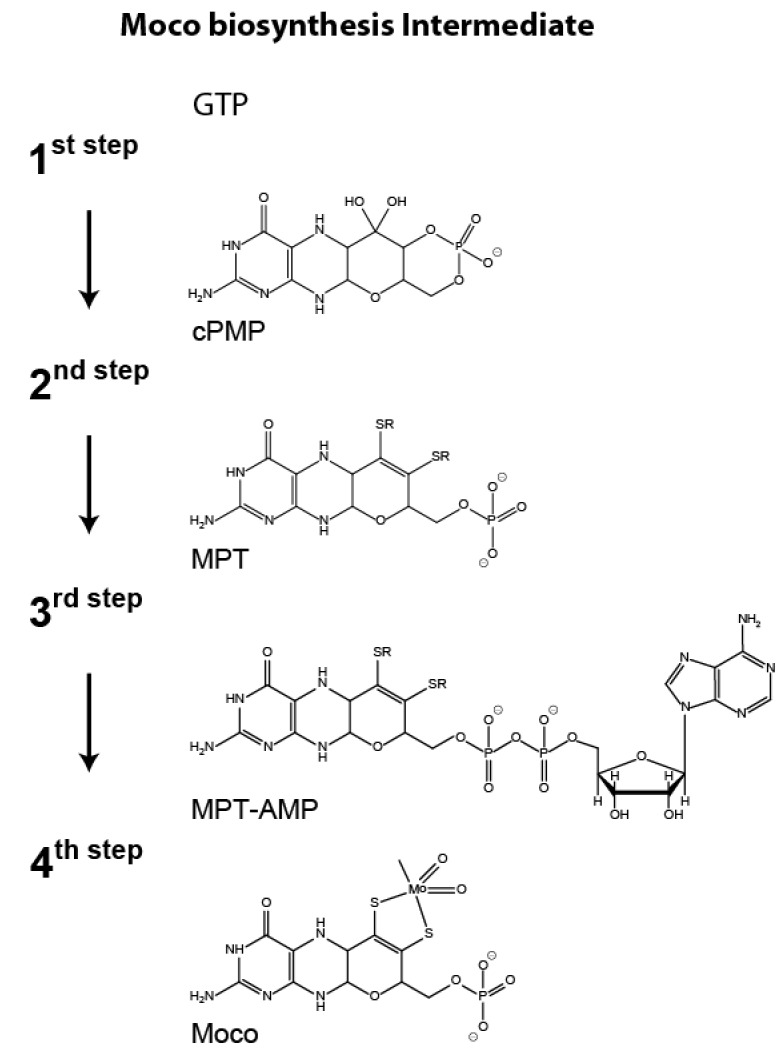
Scheme of the molybdenum cofactor biosynthesis pathway. Biosynthesis of the molybdenum cofactor (Moco) involves the stepwise conversion of guanosine triphosphate (GTP) to cyclic pyranopterin monophosphate (cPMP [8,9,16], step 1) which is converted to molybdopterin (MPT, [10,11,12]) in the following pathway step 2. In the third step, MPT is initially adenylated yielding adenylated MPT (MPT-AMP, [13,14]), which is subsequently converted to Moco [1,15] in the fourth pathway step.

In order to gain biological activity, molybdenum (Mo) needs to be coordinated by molybdopterin (MPT), resulting in the formation of the molybdenum cofactor (Moco). Moco is an essential component of vitally important enzymes found in all kingdoms of life [1]. These enzymes contribute to the biogenic nitrogen, carbon, and sulfur cycles [2]. Moco biosynthesis is an evolutionary old and highly conserved multi-step pathway which can be divided into four main steps [3]. In eukaryotes, the first step of Moco biosynthesis is generally assumed to be localized in the mitochondria of the cell [3,4]. While in plants, this step is encoded by two gene loci (*cnx2* and *cnx3*), in humans and the filamentous fungus *Neurospora crassa* (*N. crassa*), a single gene locus (*MOCS1* and *nit-7*, respectively) encodes both domains required for catalytic activity [5,6,7]. *nit-7* consists of two exons (exon 1 and exon 2) flanking the single intron 1 [7]. In the recent model, translation of the non-spliced *nit-7* transcript leads to the protein NIT-7A, which is encoded by *nit-7* exon 1 and to a small part by intron 1. Splicing of the *nit-7* mRNA results in an inactive NIT-7A domain fused to the NIT-7B domain. This variant is referred to as NIT-7AB [7]. As shown for bacterial orthologues [8,9], NIT-7A is supposed to catalyze the conversion of guanosine triphosphate (GTP) to 3′,8-cH_2_GTP, which is subsequently processed by the NIT-7B domain to cyclic pyranopterin monophosphate (cPMP). In the following, cPMP is exported into the cytoplasm of the cell [1,4] where all subsequent Moco biosynthesis steps take place [1]. In the second step of Moco biosynthesis, the dithiolene motif is introduced into cPMP, thus forming the Mo chelating scaffold molybdopterin (MPT) [10,11,12]. This reaction is performed by the heterotetrameric MPT synthase complex, which consists of two sulfur carrier subunits (*N. crassa* NIT-8) and two catalytic subunits (*N. crassa* NIT-12) [1,3,7]. As the MPT synthase catalyzes the stoichiometric conversion of cPMP to MPT, after each reaction cycle, it needs to be regenerated by the MPT synthase sulfurase (*N. crassa* NIT-1) [1,3,7]. Within steps three and four of the pathway, MPT is initially adenylated yielding adenylated MPT (MPT-AMP) [13,14], which is subsequently hydrolyzed to form Moco [15]. In *N. crassa*, these steps are catalyzed by a two domain protein encoded by *nit-9* [7]. Here, the NIT-9G domain adenylates MPT and the NIT-9E domain accepts the synthesized MPT-AMP as substrate for the subsequent molybdate insertion reaction, resulting in the formation of physiologically active Moco [1,3,7] (Figure 1).

While Moco biosynthesis has been studied extensively in prokaryotes [17,18,19] and eukaryotes [1], little is known about its regulation in eukaryotes. In this study, we took advantage of the model organism *N. crassa* in which one of the main users of Moco, *i.e.* nitrate reductase (NR; gene locus *nit-3*) [20] is under strong transcriptional control [21], which guarantees, that *nit-3* is only expressed in the presence of nitrate and under nitrogen-derepressing conditions [22]. In the present work, we characterized the transcriptional regulation of the *N. crassa* Moco biosynthesis pathway, revealing the expression of *nit-7* to be under nitrogen dependent control. Within this work, we revealed NIT-7A to be localized in the mitochondria of the cell and found that the mitochondrial import of NIT-7B requires the fusion of NIT-7A to NIT-7B. However, contrary to the present model of NIT-7 functionality [7], the NIT-7AB fusion protein was found to be degraded upon mitochondrial import, resulting in the release of NIT-7B from the fusion protein. Our new model for NIT-7 functionality was confirmed by functional complementation studies, which solidified the requirement of separate NIT-7A and a NIT-7AB derived, separate NIT-7B domain for cPMP synthesis in *N. crassa*.

## 2. Material and Methods

### 2.1. Neurospora crassa Strains and Growth Conditions

*N. crassa* strains used in this study are listed in Table 1.

Strains were grown on Vogel’s minimal medium (MM) [23] supplemented with 1.5% (*w*/*v*) sucrose as carbon source and at 30 °C. *N. crassa* liquid cultures were cultivated at 130 rpm. If required, 1.5% (*w*/*v*) agar were added to solidify media. Appropriate supplements and antibiotics were added for *N. crassa* knock out (ko) strains or strains carrying resistance markers for selection. For growth under nitrogen repressing (N-repressing) or N-derepressing and nitrate-inducing conditions [22], 75 mM ammonium chloride (NH_4_Cl) or 80 mM potassium nitrate (KNO_3_, nitrate medium) were added as sole nitrogen sources to the culture medium, respectively. Then, 300 mM sodium chlorate (NaClO_3_) were added to MM (chlorate medium) to test for catalytically active NR [24]. For crosses, synthetic cross medium (SC) was used [25]. Crossing plates were incubated at room temperature (RT) until ejected ascospores were seen. After incubating the ascospores for ~7 d in ripening buffer (0.1 M Tris-HCl pH 8.0, 2 mM EDTA) in the dark at RT, they were subsequently heat activated at 60 °C for 10 min and grown on BDES plates [26] at 30 °C overnight. Mating type determination of generated knock outs was carried out as described [27]. For race tube experiments, 3 mL solid nitrate or chlorate medium were filled in 10 mL sterile plastic serological pipets [28]. After the inoculation of 10^4^ conidia, race tubes were incubated for 72 h at 30 °C and in the dark.

### 2.2. Transformation of Neurospora crassa

*N. crassa* strains were transformed by electroporation of macroconidia as described by [29]. Homokaryotic strains were obtained either by single spore isolation or by crossing with an appropriate partner.

### 2.3. PCR-Based Cloning and Mutagenesis 

PCR-based cloning was carried out with gene-specific primers (Table S1) and Q5^®^ High-Fidelity DNA Polymerase or Phusion^®^ High-Fidelity DNA Polymerase (New England Biolabs, Ipswich, MA, USA). Obtained PCR products were subcloned using the CloneJET Cloning Kit (Thermo Fisher Scientific, Waltham, MA, USA). If indicated, nucleotide exchanges were introduced with QuikChange II (Agilent Technologies, Santa Clara, CA, USA) modified for the use of Phusion^®^ High-Fidelity DNA Polymerase [30]. Primers used for site directed mutagenesis are listed in Table S1. Plasmids were generated by restriction digest based cloning, overlap extinction PCR based cloning, or the NEBuilder^®^ HiFi DNA Assembly Cloning Kit (New England Biolabs, Ipswich, MA, USA). As shuttle vector for multiplying plasmids in *E. coli* and genomic integration of DNA fragments into the *N. crassa his-3* locus, a pCCG-C-eGFP construct or derivatives were used (Table 1) [31].

### 2.4. RNA Extraction and cDNA Synthesis

Total RNA was extracted with the *Quick*-RNA^TM^ Fungal/Bacterial Kit (Zymo, Irvine, CA, USA) followed by DNA removal using the TURBO DNA-*free*^TM^ Kit (Thermo Fisher Scientific, Waltham, MA, USA) according to the manufacturer’s instructions. RNA concentrations of the same measurement series were subsequently adjusted to identical concentrations. For cDNA synthesis, SuperScript IV reverse transcriptase (Thermo Fisher Scientific, Waltham, MA, USA) was used according to the manufacturer’s instructions. 

### 2.5. Transcript Analysis

After cDNA synthesis, Moco biosynthesis gene expression was analyzed PCR-based using gene-specific primers (Table S1) and the GoTaq^®^ DNA Polymerase (Promega, Flitchburg, WI, USA). Upon DNA gel-electrophoresis, the PCR products were documented using a ChemiDoc XRS+ imager (Bio-Rad, Hercules, CA, USA) and quantified with the Image Lab 6.0 software (Bio-Rad, Hercules, CA, USA). Quantification of *act-2* (NCU04173) cDNA served as control and reference here.

### 2.6. 5′ rapid amplification of cDNA ends

For 5′ rapid amplification of cDNA ends (5′ RACE), the SMARTer^®^ RACE 5′/3′ Kit (Takara, Kusatsu, Shiga, Japan) was used. RNA extraction and TURBO^TM^ DNase digestion was performed as described, followed by an additional RNA clean up step with the RNA Clean and Concentrator^TM^-5 Kit (Zymo, Irvine, CA, USA). Then, 1 µg total RNA was used for first strand synthesis with a *nit-7* gene-specific primer (Table S1). Touchdown PCR was carried out for the amplification of *nit-7* transcripts according to the manufacturer’s instructions. Obtained PCR products were isolated and cloned into the pRACE (Takara, Kusatsu, Shiga, Japan) or pJET vector (Thermo Fisher Scientific, Waltham, MA, USA), respectively.

### 2.7. Protein and Molybdenum Cofactor Metabolites Extraction from N. crassa

After overnight growth of *N. crassa* in liquid medium, mycelium was harvested and ground in liquid nitrogen. Subsequently, 250 µL of ice cold extraction buffer (50 mM sodium phosphate buffer, 200 mM NaCl, 5 mM EDTA, 2 % (*v*/*v*) glycerol, 5 mM glutathione, protease inhibitor (Roche cOmplete^TM^, EDTA-free), pH 7.2) were added to 100 mg ground mycelium and then incubated for 30 min on ice. Samples were mixed occasionally, followed by a centrifugation step at 21,000× *g* for 10 min at 4 °C. The obtained supernatants were transferred into fresh reaction tubes. Protein concentration was determined by Bradford protein assay (ROTI^®^-Quant, Carl Roth, Karlsruhe, BW, Germany) with bovine serum albumin serving as a concentration standard.

### 2.8. Neurospora crassa Nitrate Reductase Activity Assay

*N. crassa* NR activity was determined as described previously [32,33].

### 2.9. Generation of Monoclonal Antibodies for NIT-7A and Nitrate Reductase

The monoclonal antibodies 4C10 (anti-NIT-7A) and 5G7 (anti-NR) were generated by immunizing mice with recombinant NIT-7A or with the recombinant cytochrome *c* reducing fragment of *N. crassa* NR [33] following a standard immunization protocol. After hybridization and cloning, antibody producing hybridoma cells were screened for their binding to recombinant NIT-7A or the recombinant NR cytochrome *c* reducing fragment, respectively, by use of ELISA and Western blot analysis. Isotype analysis of the clones 4C10 and 5G7 revealed them to be an IgG1 subtype. Supernatants were grown either according to standard protocols or in serum-free medium. Both antibodies were used as culture supernatant.

### 2.10. Detection of Enhanced Green Fluorescent Protein

Recombinant eGFP fusion proteins in *N. crassa* were detected immune blot-based using an anti-green fluorescent protein mouse IgG_1_ κ monoclonal antibody (Roche, Basel, BS, Switzerland). To recognize the primary antibodies used in this study, we used Peroxidase-conjugated AffiniPure Goat Anti-Mouse IgG + IgM (H+L) (Dianova, Hamburg, Germany), which was diluted 1: 10,000 in 20 mL TBS-T and was incubated 1 h with the membrane at RT and under shaking.

### 2.11. Quantitative Detection of Molybdenum Cofactor and Its Metabolites 

Oxidation and quantification of cPMP and Moco/MPT was carried out essentially as described previously [16,34,35,36,37].

### 2.12. Bright Field Microscopy

Hyphae of strains grown on MM, nitrate, and chlorate medium were documented with the Nikon Eclipse Ni upright microscope equipped with the DS-Qi2 camera (Nikon, Shinagawa, Tokyo, Japan). The 60× Plan Apo VC (numerical aperture 1.4, oil immersion) was used as objective. Images were taken with the NIS Elements software (Nikon, Shinagawa, Tokyo, Japan) and processed using ImageJ v.1.52e (NIH, Bethesda, MD, USA).

### 2.13. Confocal Microscopy

To determine the subcellular localization of NIT-7 variants, heterokaryons of strains expressing *nit-7-egfp* variants (Table 1) and an *N. crassa* mitochondrial marker strain expressing the *atp-1* presequence fused to the mCherry coding sequence [31] were prepared as described previously [31]. Spatial localization was analyzed with the cLSM-510META scan head connected to the Axiovert 200M (Carl Zeiss, Oberkochen, BW, Germany) in a single-tracking mode. An argon laser with 488 nm (LGK 7812 ML4, Lasos) was used for eGFP excitation and fluorescence was detected by using the main dichroic beam splitter HFT UV/488/543/633 and the bandpass filter (505–530 nm) in the channel mode in front of the detector. For mCherry excitation, a helium-neon laser with 543 nm (LGK 7786P, Lasos, Jena, TH, Germany) was employed. mCherry fluorescence was detected by using the main dichroic beam splitter HFT UV/488/543/633 and a longpass filter (560 nm). When appropriate, bright field images of samples were taken with the transmitted light photomultiplier. Unidirectional sequential scanning was done with a line average of 4, 8, or 16. As objective, the 10x Plan-Neofluar (numerical aperture 0.3) and the 40× C-Apochromat (numerical aperture 1.2, water immersion) were used. Images were taken using the ZEN software (Carl Zeiss, Oberkochen, BW, Germany) and processed using ImageJ v.1.52e (NIH, Bethesda, MD, USA).

## 3. Results

**Figure 2:**
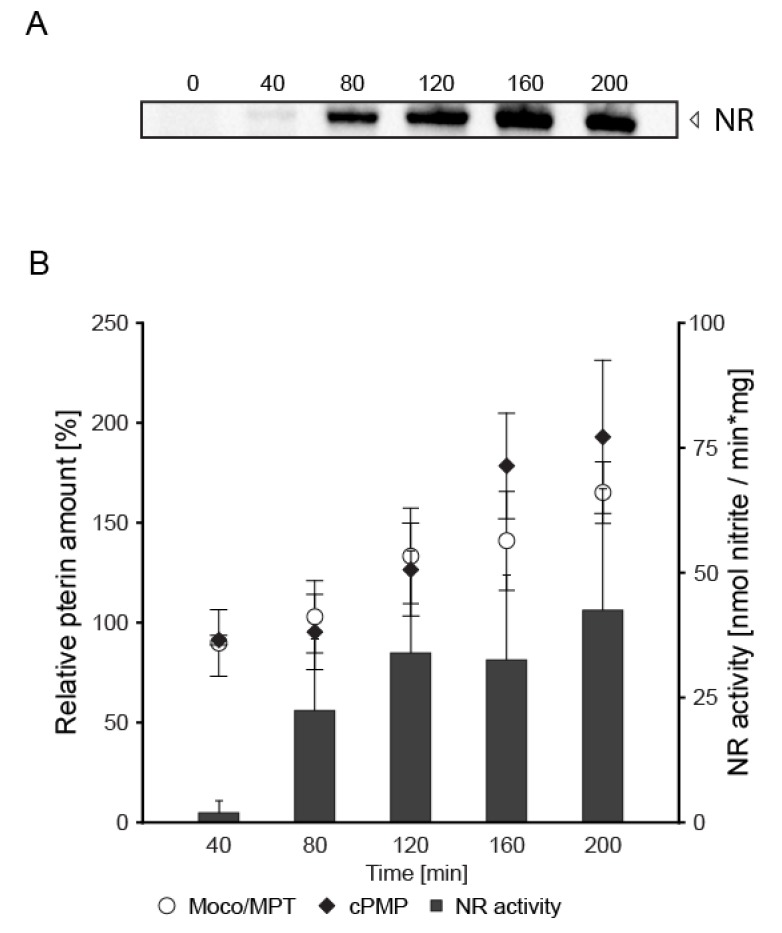
Connection of the *Neurospora crassa* Mo-metabolism with the nitrate assimilation pathway. (**A**) Western blot-based detection of nitrate reductase (NR) in *Neurospora crassa.* (*N. crassa*) crude extracts obtained at the indicated time points upon growth under nitrogen derepressing (N-derepressing) and nitrate-inducing conditions. (**B**) Quantitative detection of cyclic pyranopterin monophosphate (cPMP), molybdopterin (MPT), molybdenum cofactor (Moco), and NR activity in *N. crassa* crude extracts obtained at the indicated time points under N-derepressing and nitrate-inducing conditions. Four biological replicates were analyzed. Error bars indicate the standard deviation.

*Nitrate reductase expression is connected to Moco biosynthesis regulation*—As nitrate reductase activity depends on active site bound Moco, biosynthesis of Moco is a requirement for nitrate assimilation. However, whereas the regulation of NR is well characterized in the fungi *Aspergillus nidulans* (*A. nidulans*) and *N. crassa* [22], regulation of Moco biosynthesis is not, and notably, this also holds true for any other eukaryote. To provide first insights into the regulatory aspects behind eukaryotic Moco biosynthesis, we characterized the effect(s) of induced NR gene expression on the *N. crassa* Mo-metabolism. To do so, *N. crassa* was initially cultivated under nitrogen repressing (N-repressing) conditions and subsequently transferred to nitrate-containing media, which was depleted of any reduced N-source to establish N-derepressing and nitrate-inducing conditions [22]. Here, upon defined periods of growth, NR formation was documented Western blot based by use of a highly specific, monoclonal NR antibody, derived against the NR cytochrome *c* reducing fragment (Figure 2A).

No NR is formed in mycelia grown under N-repressing conditions (Figure 2A, 0 min), while under N-derepressing and nitrate-inducing conditions, NR becomes detectable Western blot-based at *t*_40_ (Figure 2A). Consistently, NR activity is measurable here and reaches its maximum at time point *t*_200_ (Figure 2B). The amount of NR, that is detectable by Western blot (Figure 2A), nearly correlates with the quantified NR activities (Figure 2B). Therefore, growth under N-derepressing and nitrate-inducing conditions results in the formation of enzymatically active NR. NR is a Moco dependent enzyme, and consistently the total amount of Moco/MPT (*t*_0_ = 3.7 ± 0.7 pmol per mg crude extract, *t*_200_ = 6.01 ± 0.66 pmol per mg crude extract) detectable upon induction of NR gene expression was found to correlate with the detectable NR enzymatic activity (Figure 2B). Next to Moco/MPT (Moco biosynthesis steps 2 and 3–4, Figure 1), also the Moco biosynthesis first step product cPMP can be quantified by HPLC. The quantified cPMP amounts (*t*_0_ = 32.2 ± 16.9 pmol per mg crude extract, *t*_200_ = 59.31 ± 26.44 pmol per mg crude extract) were found to correlate with the detectable Moco/MPT amounts, whereas the absolute cPMP amounts exceed the absolute Moco/MPT amounts ~ 10 fold (Figure 2B). To shed light on the underlying reason(s) for enhanced cPMP and Moco/MPT amounts, we next quantified the Moco biosynthesis gene expression strengths.

**Figure 3:**
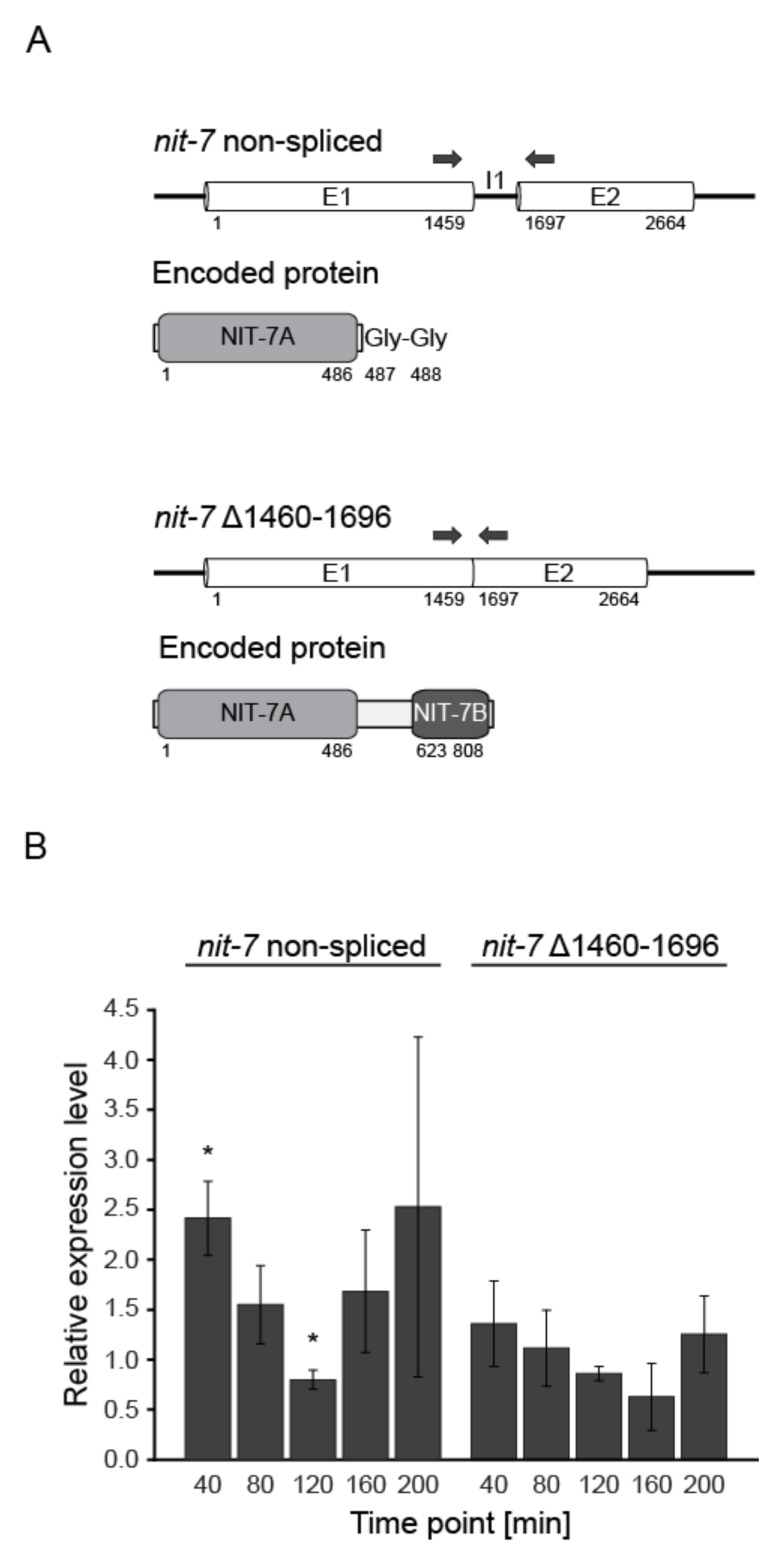
Molecular characterization of *nit-7* splice variants. (**A**) Schematic representation of the two known *Neurospora crassa nit-7* splice variants and the respective, encoded proteins [7]. Numbers indicate the nucleotide or amino acid positions, respectively. (**B**) Relative expression levels of *nit-7* non-spliced and *nit-7* Δ1460–1696 (see also Figure S1). Three biological replicates were analyzed. Error bars indicate the standard deviation and asterisks indicate significant differences compared to N-repressing conditions with *p* ≤ 0.05 according to Student’s *t*-Test.

*Transcriptional regulation of* Neurospora crassa *Moco biosynthesis*—In the following reverse transcription, PCR was employed to trace back changes of the Moco biosynthesis gene expression levels under N-derepressing and nitrate-inducing conditions. Notably, for the first step of *N. crassa* Moco biosynthesis, two functional *nit-7* transcript variants were reported [7]. To distinguish between the intron-free (*nit-7* Δ1460–1696) and the non-spliced *nit-7* variant, the primers for *nit-7* amplification were chosen to include the single *nit-7* intron (I1) which encodes for the functional relevant double glycine motif of NIT-7A. NIT-7A is encoded by exon 1 (E1), while a linker-region and NIT-7B are encoded by exon 2 (E2). The positions of the primers used for transcript quantification are indicated by arrows above the schematic representation of the *nit-7* splice variants in Figure 3A.

**Figure 4:**
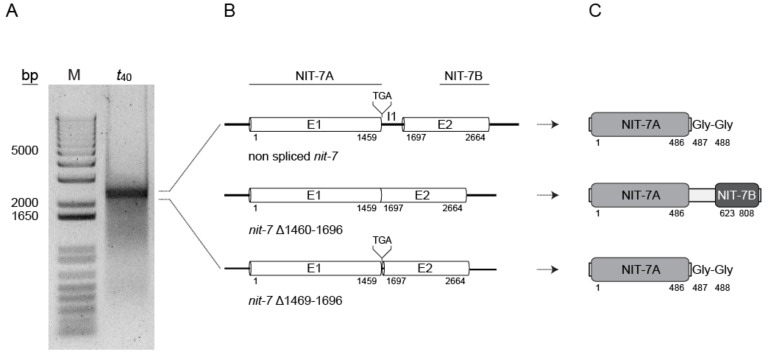
Identification of the novel *nit-7* splice variant *nit-7* Δ1469–1696. (**A**) Results from 5′ RACE (5′ rapid amplification of cDNA ends). (**B**) Schematic representation of the *nit-7* exon (E) / intron (I) structure of non-spliced *nit-7*, splice variants *nit-7* Δ1460–1696 and *nit-7* Δ1469–1696, respectively. Numbers below indicate the nucleotide position of the exon / intron borders. When existent in the respective *nit-7* mRNA, the stop codon within intron 1 is indicated above. (**C**) Schematic representation of the encoded NIT-7 domains. The first and last amino acids of NIT-7A and NIT-7B are given below. The functional relevant double glycine motif encoded by I1 and the linker region encoded by exon 2 (white bar) are indicated. Numbers refer to the amino acid position within NIT-7.

Amongst all transcripts analyzed (Figure S1), exclusively the amount of the non-spliced *nit-7* mRNA (encoding NIT-7A) was found to be significantly enhanced under N-derepressing and nitrate-inducing conditions (Figure 3B). Therefore, enhanced Moco demand results in an increase of the non-spliced *nit-7* transcript and hence the regulation of *nit-7* splicing.

nit-7 *splice variants*—The human *nit-7* homolog *MOCS1* encodes for multiple splice variants with differences in the 5′- and 3′- ends [6,38,39,40]. To test whether or not also yet unidentified *nit-7* splice variants contribute to fungal cPMP synthesis and are potentially subject to transcriptional regulation, we carried out a 5′ RACE (5′ rapid amplification of cDNA ends) experiment using mycelium grown under N-derepressing and nitrate-inducing conditions (*t*_40_, see also Figure 2). 5′ RACE yielded products sized ~2.5 kb (Figure 4). These were sequenced, revealing that the two known *nit-7* splice variants (non-spliced *nit-7* and *nit-7* Δ1460–1696, see Figure 3A for comparison) were not separated by gel electrophoresis but were detected as one band. Next to these, a novel yet unknown splice variant *nit-7* Δ1469–1696 was identified by sequencing (Figure 4), whose relevancy for Moco biosynthesis has been studied by us in the following.

**Figure 5:**
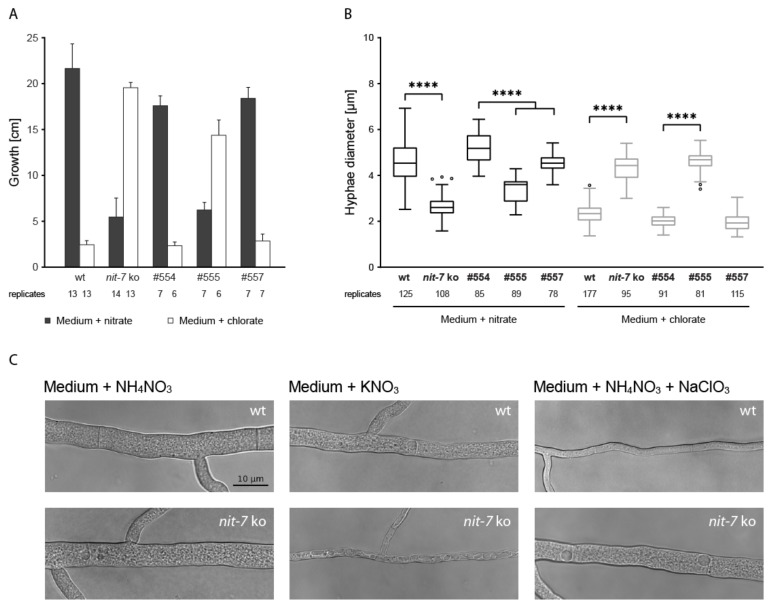
Quantification of the *Neurospora crassa nit*-phenotype. (**A**) Nitrate-dependent growth (i.e., growth under nitrogen-derepressing and nitrate-inducing conditions = nitrate medium) of indicated *N. crassa* strains, see Table 1, wt = wild type. In the complementary experimental setup, growth was quantified on medium containing chlorate. (**B**) The hyphal diameter of the strains analyzed in (A) has been determined and is shown as Tukey´s box blots. Multiple comparisons analysis was carried out using ordinary one-way ANOVA (Tukey´s method) with **** *p* ≤ 0.001. (**C**) Representative images of hyphae from strains assayed in (A+B) on different media.

*The* Neurospora crassa nit-*phenotype*—In the available literature, e.g., [7,22,41] *N. crassa nit*-strains are described to lack the nitrate utilization capability (*nit* is the abbreviation for nitrate non utilizer) and next to this rather qualitative description, no quantitative data describing the *nit*-phenotype is available so far. To document any impact of the novel splice variant *nit-7* Δ1469–1696 on *N. crassa* Moco biosynthesis, we established quantitative methods which aimed to provide the experimental basis for any further phenotypic characterization of the *N. crassa* Moco biosynthesis. Therefore, we initially quantified the hyphal growth of the *nit-7* knock out (ko) strain (Table 1, Figure 5) [7] and unexpectedly we found this strain to possess residual growth under N-derepressing and nitrate-inducing conditions (nitrate-containing medium) (wildtype (wt) = 21.7 ± 2.7 cm, *nit-7* ko strain = 5.5 ± 1.0 cm), although significant growth on chlorate-containing medium was detected (wt = 2.4 ± 0.4 cm, *nit-7* ko strain = 19.6 ± 0.6 cm), (Figure 5C).

As growth of any *nit*-strain on chlorate-containing medium naturally excludes growth on nitrate-containing medium [24], we next went on to identify the underlying reason behind this observation. We found that the *nit-7* ko strain possesses a reduced lateral spreading as compared to the wt strain when grown on nitrate-containing medium. Furthermore, the diameter of primary hyphae of both, the wt and the *nit-7* ko strain grown on nitrate and on chlorate-containing medium was quantified (Figure 5B), respectively. When grown on nitrate-containing medium, we identified *nit-7* ko strain hyphae to possess a significantly reduced diameter and an enhanced number of vacuoles as compared to the wt strain (Figure 5B). However, when the *N. crassa nit-7* ko strain was grown on chlorate-containing medium, the hyphae diameter and vacuole number was comparable to that documented for the *N. crassa* wt strain cultivated on nitrate-containing medium.

We conclude, that when cultivated on nitrate-containing medium, the *nit-7* ko strain metabolizes endogenously available N-sources which—combined with (i) reduced lateral spreading, (ii) a reduction of the hyphae diameter, and (iii) an increase of the vacuole number—best explains the detected linear growth here.

*Relevancy of splice variant* nit-7 *Δ1469–1696 for Moco biosynthesis*—Upon establishing quantitative methods to characterize the *N. crassa nit*-phenotype, we were able to characterize the relevancy of *nit-7* splice variant Δ1469–1696 for *N. crassa* Moco biosynthesis. For this, *N. crassa* strain #557 (Table 1), expressing the *nit-7* variant incompetent of producing *nit-7* Δ1469–1696 ectopically in a *nit-7* ko background [7] (Table 1, Figure 5A) was created. For comparison, *N. crassa* strain #555 (Table 1) was generated, ectopically expressing the *nit-7* variant incompetent of producing *nit-7* Δ1460–1696 again in the *nit-7* ko background [7].

Using the above described quantitative methods, we assayed strains #557 and #555, whereby we also established a control strain expressing wt *nit-7* ectopically in the *nit-7* ko background [7]. As expected, strain #554 displays significant growth on nitrate-containing medium (17.6 ± 1.1 cm), comparable to that of the wt strain (21.7 ± 2.7 cm) and also hyphal diameters were found to be wt like (wt strain = 4.6 ± 0.9 µm, #554 = 5.2 ± 0.7 µm). On the contrary, strain #555 (expressing the *nit-7* variant incompetent of producing *nit-7* Δ1460–1696) does not possess significant growth. Strain #557 (expressing the *nit-7* variant incompetent of producing *nit-7* Δ1469–1696) shows wt like growth behavior except for reduced hyphae diameter on nitrate-containing medium (Figure 5B). When grown on chlorate-containing medium, all strains showed the complementing phenotype expected from growth on nitrate-containing medium. Hence, we conclude that the novel *nit-7* splice variant Δ1469–1696 is not essential for Moco biosynthesis.

**Figure 6:**
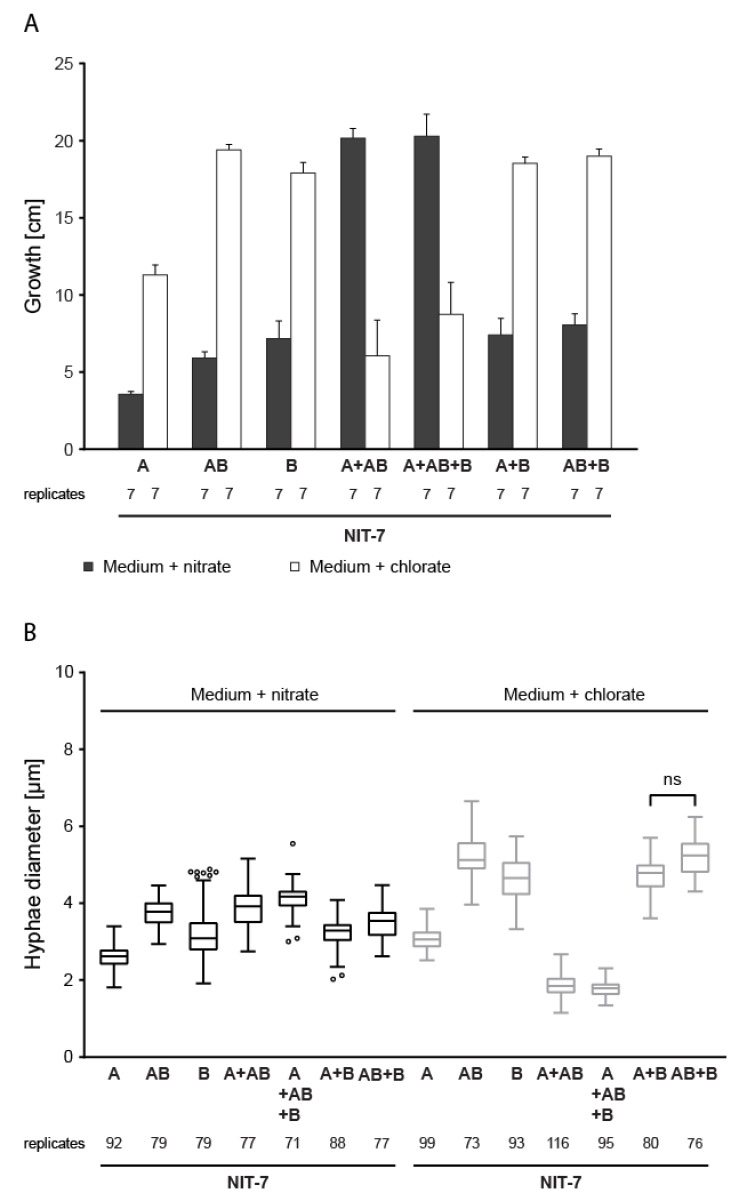
Functional complementation of the *Neurospora crassa nit-7* mutant strain. (**A**) *Neurospora crassa nit-7* knock out strains ectopically expressing either *nit-7a*, *nit-7b*, or *nit-7ab* (Table 1) were generated and grown as heterokaryons under nitrogen derepressing and nitrate-inducing conditions (nitrate-containing medium) and on chlorate-containing medium, respectively. (**B**) The hyphal diameter of the strains analyzed in (**A**) has been determined and is shown as Tukey´s box plots. Multiple comparisons analysis was carried out using ordinary one-way ANOVA (Tukey´s method). Differences, which are not significant, are indicated (ns).

*The NIT-7 functional domains*—As cPMP synthesis depends on both NIT-7 domains, the contribution of domains A, B, and the AB domain fusion to *N. crassa* cPMP synthesis was deciphered. The *A. nidulans nit-7* homolog *cnxABC* was discussed to be expressed as bicistronic transcript, translating both catalytic domains separately [42]. In line with this, the *nit-7* exon 2 harbors a potential start codon (http://atgpr.dbcls.jp/) [43], which encodes for the first residue of NIT-7B (M623). To test for a possible function of a separate NIT-7B domain starting with M623, three novel *N. crassa* strains within the *nit-7* ko background [7] were created, each constitutively expressing either NIT-7A (*N. crassa* strain #552), NIT-7B (#558), or NIT-7AB (#553), respectively. The functional interplay of the respective NIT-7 domains was subsequently assayed by heterokaryon growth (Figure 6), which has been quantified as described above.

This setup revealed that growth on nitrate-containing medium depends on the presence of both, NIT-7A and NIT-7AB in the heterokaryon. Notably, wt-like growth of the heterokaryons formed by strains expressing NIT-7A plus NIT-7B or NIT-7AB plus NIT-7B was not detectable. As plant and mammalian cPMP synthesis was reported to be localized in the mitochondria of the cell [4,44], we determined the subcellular localization of the fungal NIT-7A, NIT-7AB, and NIT-7B domain.

**Figure 7:**
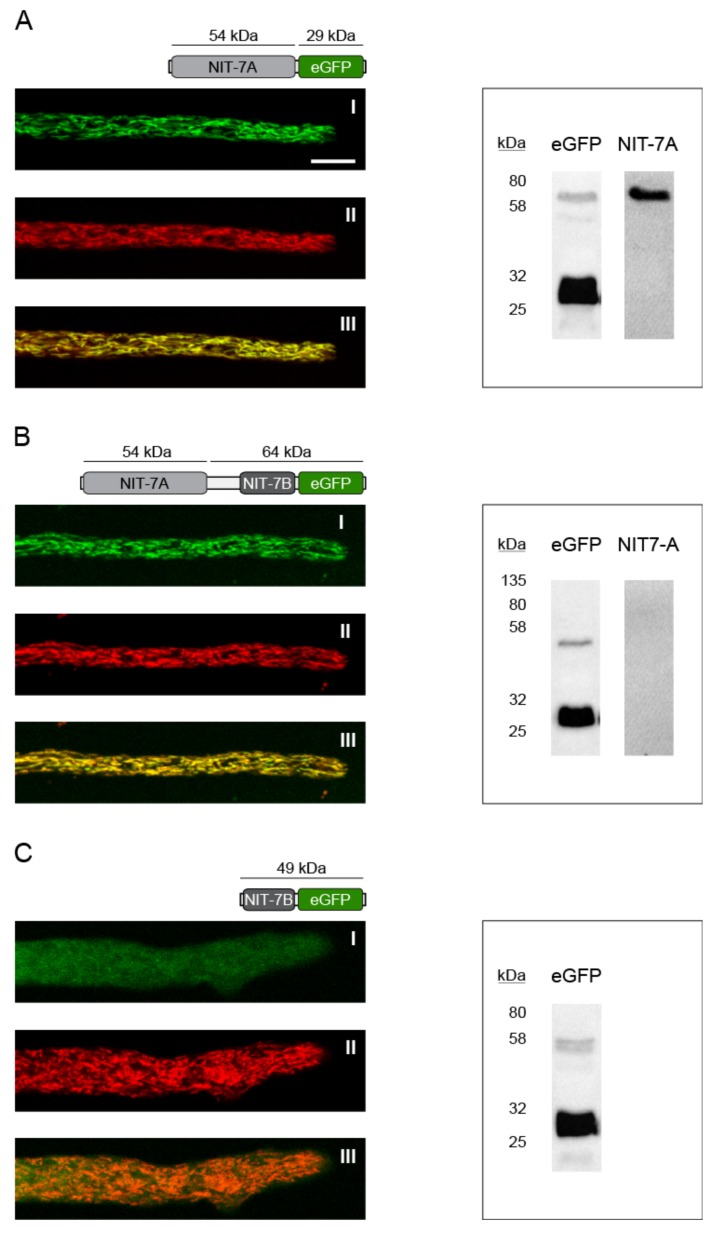
Localization of NIT-7. (A I) Subcellular localization of the NIT-7A-eGFP fusion protein, (**A** II) subcellular localization of ATP-1-Presequence-mCherryNC [31], (**A** III) overlay of the signals obtained for I and II. (**B** I) Subcellular localization of the NIT-7AB-eGFP fusion protein, (**B** II) subcellular localization of ATP-1-Presequence-mCherryNC, (**B III**) overlay of the signals obtained for I and II. (**C** I) Subcellular localization of the NIT-7B-eGFP fusion protein, (**C** II) subcellular localization of ATP-1-Presequence-mCherryNC, (**C** III) overlay of the signals obtained for I and II. (**A**) Scale bar = 10 µm. Scale bars for (**B**) and (**C**) were not shown but are identical to that shown in (**A**). The corresponding immuno-blots are shown next to the results from the live cell imaging approach, with the used antibodies indicated. For immune blotting, 20 µg of crude extracts prepared from the analyzed strains were used. The primary and secondary antibodies used are described in detail in the Materials and Methods section.

*Localization of NIT-7 domains*—To determine the subcellular localization of NIT-7A, NIT-7B, and NIT-7AB, transgenic *N. crassa* strains expressing the respective C-terminal eGFP fusions were created (Table 1). NIT-7A- and NIT-7AB-eGFP fusions localized to the mitochondria (Figure 7A,B), while in contrast, NIT-7B did not (Figure 7C).

Recent work carried out in the human system reported that cytosolic cleavage of the NIT-7AB homologous protein MOCS1AB exposes an internal mitochondrial targeting signal, affecting the mitochondrial import of thus liberated MOCS1B [44]. To test for the putative cytosolic cleavage and subsequent mitochondrial import of NIT-7B, we created an *N. crassa* strain expressing a *nit-7ab-egfp* variant, lacking the mitochondrial targeting signal encoding sequence. This truncated NIT-7AB-eGFP fusion protein was shown to reside in the cytosol of the cell, while no mitochondrial import of NIT-7B eGFP was detectable (Figure S2). Therefore, we conclude that the mammalian import model for the first step Moco biosynthesis proteins [44] does not hold true for *N. crassa* NIT-7.

While in *N. crassa*, no cytosolic release of NIT-7B from the AB domain fusion was detectable, we identified the NIT-7AB-eGFP fusion (possessing the N-terminal mitochondrial targeting signal) to lack the A-domain after import into the mitochondria (Figure 7B). This finding was documented by the fact that the mitochondrial localized NIT-7B-eGFP fusion was detectable immunoblot-based while the A-domain was not (Figure 7B). We conclude that upon mitochondrial import, NIT-7B is released from NIT-7AB. Therefore, the current model of NIT-7 functionality [7] requires revision.

## 4. Discussion

**Figure 8:**
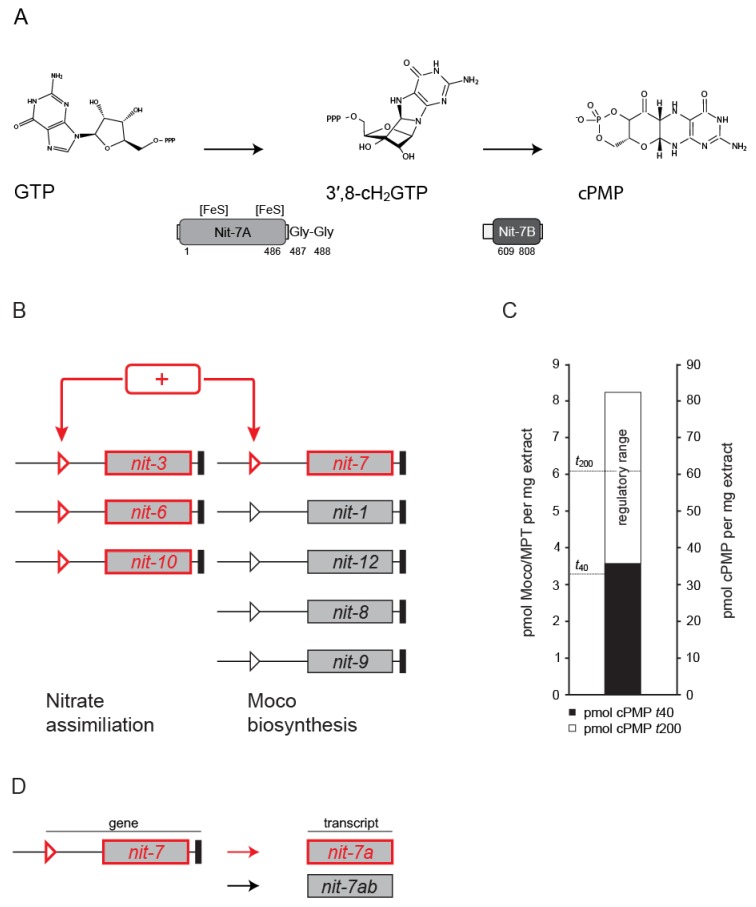
Co-regulation of *Neurospora crassa* molybdenum cofactor biosynthesis and nitrate assimilation. (**A**) Schematic reaction detail of the first step of molybdenum cofactor (Moco) biosynthesis, where GTP is sequentially processed yielding 3´,8-cH_2_GTP as intermediate and cyclic pyranopterin monophosphate (cPMP) as reaction product. Here, NIT-7A is suggested to act as 3´,8-cH_2_GTP cyclase, whereby NIT-7B is assumed to function as cPMP synthase [8,9]. (**B**) Schematic representation of the interlaced regulation of the Moco biosynthesis and nitrate assimilation pathway in *Neurospora crassa*. + = nitrogen derepressing (N-derepressing) and nitrate-inducing conditions. Triangles indicate the promotors, black boxes the terminators of the genes indicated. (**C**) Quantitative data for cellular Moco/molybdopterin (MPT) and cPMP amounts at *t*_40_ and *t*_200_ (single biological experiment, see Figure 2). The dashed lines indicate the Moco/MPT synthesis implemented at *t*_40_ and *t*_200_, respectively. The regulatory range is defined as *t*_200_–*t*_40_. (**D**) Detail of the regulatory scheme shown in (B). Under N-derepressing and nitrate-inducing conditions (red arrow), the non-splice *nit-7* transcript (encoding NIT-7A, see A) is increasingly expressed.

Synthesis of Moco involves the stepwise conversion of GTP into the known Moco metabolites 3′,8-cH_2_GTP [8,9], cPMP [16] (Figure 8A), MPT [10,11,12], MPT-AMP [13,14], and finally Moco [1,45,46,47]. Numerous Moco biosynthesis enzymes are required for its synthesis [1,3], which demands the precise coordination of the expression strengths of all encoding genes. In our work, we identified the tight regulation of the (Moco-dependent) NR in *N. crassa* to be interlaced with the Moco biosynthesis pathway on the transcriptional level. Under nitrogen derepressing (N-derepressing) and nitrate-inducing conditions, expression of nitrate reductase (*nit-3*) [20], nitrite reductase (*nit-6*) [48], and the nitrate importer (*nit-10*) [49] is up-regulated (Figure 8B). In our work, we identified the amounts of the non-spliced NIT-7A encoding *nit-7* gene transcript (Figure 8D) to be likewise enhanced under these conditions, while the expression strengths of the remaining Moco-biosynthesis genes were not up-regulated. This finding underlines the results of [50], who suggest that in *Escherichia coli*, the NIT-7A homolog MoaA catalyzes the rate-determining step in cPMP synthesis (Figure 8A), as documented by the finding that the *k_cat_* for the conversion of 3´,8-cH_2_GTP into cPMP is approximately 4-fold higher than the *k_cat_* determined for the conversion of GTP to 3´,8-cH_2_GTP [8,9]. 

However, to our surprise, the absolute HPLC-based pterin quantification revealed that in *N. crassa*, cPMP amounts are highly elevated as against Moco/MPT amounts at any time point in the experimental series (Figure 2B, Figure 8C). We conclude, that cPMP is not quantitatively converted into MPT— neither under N-repressing nor N-derepressing and nitrate-inducing conditions. Nonetheless, the cPMP to Moco/MPT ratio increased in a time-dependent fashion; evidently, *nit-7* expression ultimately leads to enhanced cPMP production. As enhanced amounts of cellular cPMP goes hand in hand with the formation of enhanced Moco/MPT amounts and enhanced NR enzymatic activity, we conclude that steps two and three–four of the pathway provide a constant cPMP conversion capacity, which is only fully used when the cellular cPMP amount is maximal (Figure 8C). Consistently, our transcript quantification approach identified the amount of the remaining Moco-biosynthesis transcripts to be not significantly enhanced under N-derepressing and nitrate-inducing conditions (Figure 8B). Transcriptional regulation results in the enhanced formation of non-spliced *nit-7* transcript (Figure 8D), which encodes the mitochondrial localized NIT-7A domain. However, for cPMP synthesis, the NIT-7B domain likewise requires mitochondrial import.

Most interestingly here, the NIT-7AB fusion was found to undergo proteolytic cleavage after mitochondrial import, which results in the degeneration of the A-domain and the liberation of NIT-7B. This finding is contrary to that reported for the mammalian system where native MOCS1AB was found to undergo proteolytic cleavage, thus exposing an internal mitochondrial import signal required for the mitochondrial import of the B-domain [44]. Therefore, in both—mammals and fungi—other than postulated earlier [7,51], functionality of the first Moco biosynthesis step essentially depends on the presence of separated A- and B-domains in the mitochondria. The existence of different pathways in mammals and fungi, which both result in the B-domain release from the AB precursor, allows it to conclude that these have evolved after the fungal and animal lineage diverged from their last common ancestor [52].

## Figures and Tables

**Table 1:** *Neurospora crassa* strains used in this work. Next to the strain number, for P*ccg-1* driven genes, the encoded protein product is given in parenthesis. mts: mitochondrial targeting sequence. eGFP: enhanced green fluorescent protein.

Strain Number	Genotype	Origin
FGSC 2489	74-OR23-1V; mat A	FGSC
FGSC 4317	fl; mat A	FGSC
FGSC 4347	fl; mat a	FGSC
FGSC 9720	his-3-; Δmus-52::bar+; mat A	FGSC
G200 (ATP-1-Preseq-mCherryNC)	his-3::Pccg1-atp1.1_120-mcherry; Δmus-52::bar+; mat A	Wirsing et al., 2015
G253	Δmus-52::bar+; Δnit-7::hph+; mat a	Probst et al., 2014
G546	his-3-; Δmus-52::bar+; Δnit-7::hph+; mat A	This study
G554	his-3::nit-7+(EC); Δmus52::bar+; Δnit-7::hph+; mat A	This study
G555	his-3::nit-7.1461T>C(EC); Δmus52::bar+; Δnit-7::hph+; mat A	This study
G557	his-3::nit-7.1470T>C(EC); Δmus52::bar+; Δnit-7::hph+; mat A	This study
G552 (NIT-7A)	his-3::Pccg1-nit-7.1_1467; Δnit-7::hph+; Δmus-52::bar+; mat A	This study
G553 (NIT-7AB)	his-3::Pccg1-nit-7.1_1459+1697_2664; Δnit-7::hph+; Δmus-52::bar+; mat A	This study
G558 (NIT-7B)	his-3::Pccg1-nit-7.2104_2664; Δnit-7::hph+; Δmus-52::bar+; mat A	This study
G542 (NIT-7A-eGFP)	his-3::Pccg1-nit-7.1_1464-egfp; Δmus-52::bar+; mat A	This study
G544 (NIT-7AB-eGFP)	his-3::Pccg1-nit-7.1_1459+1697_2661-egfp; Δmus-52::bar+; mat A	This study
G550 (NIT-7B-eGFP)	his-3::Pccg1-nit-7.2104_2661-egfp; Δmus-52::bar+; mat A	This study
G575 (NIT-7A without mts-eGFP)	his-3::Pccg1-nit-7.163_1464-egfp; Δmus-52::bar+; mat A	This study
G576 (NIT-7AB without mts-eGFP)	his-3::Pccg1-nit-7.163_1459+1697_2661-egfp; Δmus-52::bar+; mat A	This study
G577 (Linker-NIT-7B-eGFP)	his-3::Pccg1-nit-7.1459+1697_2661-egfp; Δmus-52::bar+; mat A	This study

## Supplementary Materials

Supplementary materials can be found at https://www.mdpi.com/2076-2607/8/4/534/s1.
