# Supplementary material for: The First Step of Neurospora crassa Molybdenum Cofactor Biosynthesis: Regulatory Aspects under N-Derepressing and Nitrate-Inducing Conditions
Source: Microorganisms. 2020 Apr 7;8(4):534. doi: 10.3390/microorganisms8040534 (PMC7232280; doi:10.3390/microorganisms8040534)
Supplement: Supplementary file 1 [file microorganisms-08-00534-s001.pdf]

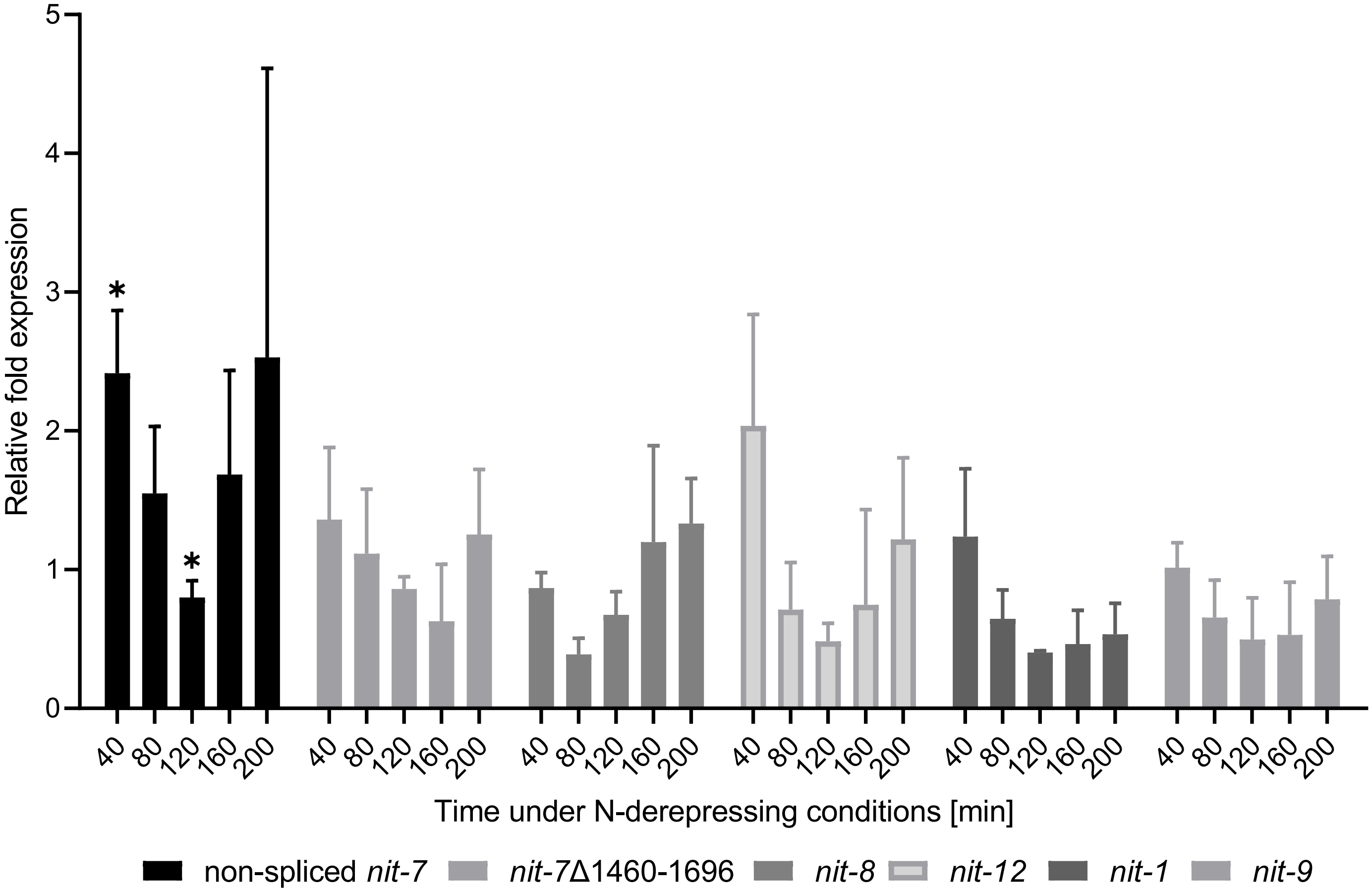

Figure S1: Relative expression levels of molybdenum cofactor biosynthesis genes. Three biological replicates were analyzed. Error bars indicate the standard deviation and asterisks indicate significant differences of expression strengths as compared to nitrogen repressing conditions with $P \leq 0.05$ according to Student's *t*-Test.

Figure -S1-

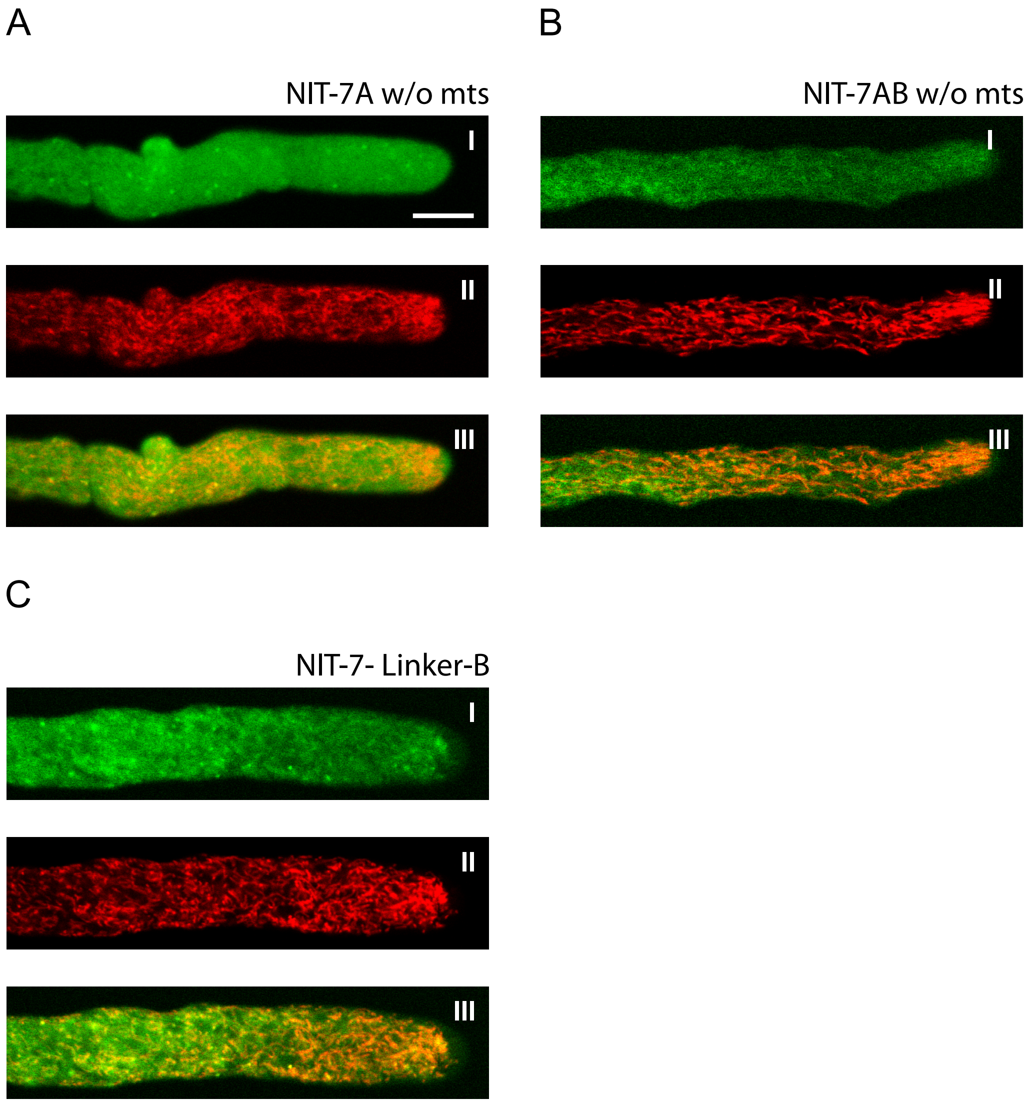

Figure S2: Localization of NIT-7. (A I) Subcellular localization of the NIT-7A-eGFP fusion protein lacking the mitochondrial targeting signal (mts), (A II) subcellular localization of ATP-1-Presequence-mCherryNC [35], (A III) overlay of the signals obtained for I and II. Scale bar indicates 10 µm. (B I) Subcellular localization of the NIT-7AB-eGFP fusion protein lacking the mts, (B II) subcellular localization of ATP-1-Presequence-mCherryNC, (B III) overlay of the signals obtained for I and II. (C I) Subcellular localization of the NIT-7-Linker-B-eGFP fusion protein derived from the translation of the entire *nit-7* exon 2 fused in frame to the eGFP encoding sequence, (C II) subcellular localization of ATP-1-Presequence-mCherryNC, (C III) overlay of the signals obtained for I and II. Scale bars for (B) and (C) were not shown but are identical to that shown in (A).

Figure -S2-

Table S1: Primer used in this work.

Name	Sequence (5‘ - 3‘ direction)	Purpose
1050	CTGAGGGTATTGCTGGTGAGAG	Amplification of *nit-7* after RT-PCR
1053	CTCCACCGACGGGACTTCTC	Amplification of *nit-7* after RT-PCR
1231	CAATCAGCTCTGTCCCTCGG	Amplification of *nit-1* after RT-PCR
1232	GAAGGTGGGTGATGAGGGAC	Amplification of *nit-1* after RT-PCR
1233	TGATCCCTTTGCGTTGGAGA	Amplification of *nit-8* after RT-PCR
1234	CGTTATCCTCGCCGTCTTCA	Amplification of *nit-8* after RT-PCR
1235	GGCAACTCAACAACCAACCC	Amplification of *nit-12* after RT-PCR
1236	GGTGAGAATGGAGCGGCATA	Amplification of *nit-12* after RT-PCR
1237	AGCCTACCACGTTTGCTACC	Amplification of *nit-9* after RT-PCR
1238	TCTCTGTCCTCCTGTGCTGA	Amplification of *nit-9* after RT-PCR
1083	CTCTCAGGTACCCCATCGAG	Amplification of *act-2* after RT-PCR
1194	GTAGATGGGGACGACGTGAG	Amplification of *act-2* after RT-PCR
1291	GATTACGCCAAGCTTGGCCCTCTCCAAATCTTCTGACCCCGCA	5'RACE of *nit-7* (pRACE vector)
1659	GGCCCTCTCCAAATCTTCTGACCCCGCA	5'RACE of *nit-7* (pJET vector)
810	CCAGCGTCGGTTTCTTGTGCTTAC	Amplification of *nit-7*
811	AAATGGATGGGAGCCGCACGCA	Amplification of *nit-7*
1660	GCTGTCTTTGAACTGAGTGACTTCTGGTAGCTGC	NEBuilder assembly of *nit-7* with flanking regions in pCCG (DNA #698, *N. crassa* #554)
1661	CACTCAGTTCAAAGACAGCCAACTTGAGACCG	NEBuilder assembly of *nit-7* with flanking regions in pCCG (DNA #698, *N. crassa* #554)
1662	GAAGTAGCGCTGCCTACCTAGC	NEBuilder assembly of *nit-7* with flanking regions in pCCG (DNA #698, *N. crassa* #554)
1663	TAGGCAGCGCTACTTCTAAGGAATGTCTGAGGTGCAC	NEBuilder assembly of *nit-7* with flanking regions in pCCG (DNA #698, *N. crassa* #554)
1664	CCCCGGGAGGTAGGCGTGATAAGAAATGGATGG	NEBuilder assembly of *nit-7* with flanking regions in pCCG (DNA #698, *N. crassa* #554)
1665	GCCTACCTCCCGGGGTGGCATCCC	NEBuilder assembly of *nit-7* with flanking regions in pCCG (DNA #698, *N. crassa* #554)
1614	GAGCACTTTTAAAGTTCTGCTATGTGGC	Split amp^{R}-cassette of pCCG for NEBuilder assembly
1621	GCAGAACTTTAAAAGTGCTCATCATTGGAAA	Split amp^{R}-cassette of pCCG for NEBuilder assembly
1783	GGGTGATGCTCGTCCTCCTC	Insertion of mutation *nit-7*.1470T>C in DNA #698 with NEBuilder (*N. crassa* #557)
1784	AGGACGAGCATCACCCACCA	Insertion of mutation *nit-7*.1470T>C in DNA #698 with NEBuilder (*N. crassa* #557)
1514	CGAACATCACCCGCCAATCAAGATCATTGGCCG	Insertion of mutation *nit-7*.1461T>C in DNA #698 with QuikChange II (*N. crassa* #555)
1515	GATCTTGATTGGCGGGTGATGTTCGTCCTCCTC	Insertion of mutation *nit-7*.1461T>C in DNA #698 with QuikChange II (*N. crassa* #555)
2001	GAAGCCATTCGCGATTTGGTTGATGTGAGG	NEBuilder assembly of nit-7 in pCCG for expression of NIT-7A- and NIT-7AB-eGFP fusions without mts (*N. crassa* #575 and #576)
2004	TGGTGGGGGAGGAATGTCCGCCTGG	NEBuilder assembly of nit-7 in pCCG for expression of NIT-7A-eGFP fusion without mts (*N. crassa* #575)
2006	ATTCCTCCCTCCCCATCCTG	NEBuilder assembly of nit-7 in pCCG for expression of NIT-7AB-eGFP fusion without mts (*N. crassa* #576)
1327	TTGGATCCATGGTCTCCATAACCACCAAG	Amplification of *nit-7*.2104_2661 for insertion into pCCG via restriction digest (NIT-7B-eGFP, *N. crassa* #550; NIT-7B, N. crassa #558)
1329	AAACTAGTCTCCCCATCCTGTCTCC	Amplification of *nit-7*.2104_2661 for insertion into pCCG via restriction digest (NIT-7B-eGFP, *N. crassa* #550)
2108	ATTCCTCCCTCCCCATCCTG	NEBuilder assembly of *nit-7* exon 2 in pCCG (*N. crassa* #577)
2109	TGGGGAGGGAGGAATGTCC	NEBuilder assembly of *nit-7* exon 2 in pCCG-C (*N. crassa* #577)
1278	TATA AGATCT ATGTCTGCTGTGGCCCG	Amplification of nit-7 exon 1 for insertion into pCCG via restriction digest (NIT-7A, *N. crassa* #552)
1279	ATATCACGTG TCACCCACCAATCAAGATCATTG	Amplification of nit-7 exon 1 for insertion into pCCG via restriction digest (NIT-7A, *N. crassa* #552)
1341	TATACTCGAGATGTCTGCTGTGGCCC	Amplification of *nit-7* exon 1 for fusion PCR with exon 2 and insertion into pCCG via restriction digest (NIT-7AB, *N. crassa* #553)
1342	CTGAAATATAGCATTGGTATTGGTATCAATCAAGATCATTGGCC	Amplification of *nit-7* exon 1 for fusion PCR with exon 2 and insertion into pCCG via restriction digest (NIT-7AB, *N. crassa* #553)
1343	GGCCAATGATCTTGATTATACCAATACCAATGCTATATTTCAG	Amplification of *nit-7* exon 2 for fusion PCR with exon 1 and insertion into pCCG via restriction digest (NIT-7AB, *N. crassa* #553)
1344	TATAAGATCTCTCCCCATCCTGTCTCC	Amplification of *nit-7* exon 2 for fusion PCR with exon 1 and insertion into pCCG via restriction digest (NIT-7AB, *N. crassa* #553)
1328	AAACTAGTTTACTCCCCATCCTGTCTC	Amplification of *nit-7*.2104_2661 for insertion into pCCG via restriction digest (NIT-7B, *N. crassa* #558)
